# Cabozantinib‐nivolumab sequence in metastatic renal cell carcinoma: The CABIR study

**DOI:** 10.1002/ijc.34126

**Published:** 2022-06-06

**Authors:** Yann‐Alexandre Vano, Letuan Phan, Gwenaelle Gravis, Iphigénie Korakis, Friederike Schlürmann, Denis Maillet, Mostefa Bennamoun, Nadine Houede, Delphine Topart, Delphine Borchiellini, Philippe Barthelemy, Raffaele Ratta, Thomas Ryckewaert, Ali Hasbini, Sophie Hans, Sheik Emambux, Sandra Cournier, Elena Braychenko, Réza‐Thierry Elaidi, Stéphane Oudard

**Affiliations:** ^1^ Medical Oncology, Hôpital Européen Georges Pompidou AP‐HP Centre—Université Paris Cité Paris France; ^2^ INSERM U970, PARCC Paris France; ^3^ Centre de Recherche des Cordeliers, INSERM Université Paris Cité, Sorbonne Université Paris France; ^4^ ARTIC—Association pour la Recherche de Thérapeutiques Innovantes en Cancérologie, Hôpital Européen Georges Pompidou, AP‐HP Centre Paris France; ^5^ Medical Oncology, Institut Paoli‐Calmettes Aix‐Marseille University, CRCM Marseille France; ^6^ Medical Oncology, Institut Universitaire du Cancer—Toulouse—Oncopole Toulouse France; ^7^ Medical Oncology, Centre Hospitalier Intercommunal, Quimper Quimper France; ^8^ Medical Oncology, IMMUCARE, Centre Hospitalier Lyon Sud Institut de Cancérologie des Hospices de Lyon (IC‐HCL) Pierre‐Bénite France; ^9^ Medical Oncology, Institut Mutualiste Montsouris Paris France; ^10^ Medical Oncology, Institut de cancérologie du Gard, Nimes Montpellier University France; ^11^ Medical Oncology, Hopital Saint‐Eloi (CHU de Montpellier) Montpellier France; ^12^ Medical Oncology, Centre Antoine Lacassagne Université Côte d'Azur Nice France; ^13^ Medical Oncology, Institut de Cancérologie Strasbourg Europe Strasbourg France; ^14^ Medical Oncology, Hopital Foch Suresnes France; ^15^ Medical Oncology, Centre Oscar Lambret Lille France; ^16^ Medical Oncology, Clinique Pasteur Lanroze Brest France; ^17^ Department of Medical Oncology, Hôpital Henri‐Mondor AP‐HP—Université de Paris Est Créteil France; ^18^ Medical Oncology Centre Hospitalier Universitaire Poitiers Poitiers France

**Keywords:** cabozantinib, immunotherapy, matching‐adjusted study, nivolumab, renal cell carcinoma, tyrosine kinase inhibitor

## Abstract

Nivolumab and cabozantinib are approved agents in mRCC patients after sunitinib/pazopanib (TKI) failure. However, the optimal sequence, cabozantinib then nivolumab (CN) or nivolumab then cabozantinib (NC), is still unknown. The CABIR study aimed to identify the optimal sequence between CN and NC after frontline VEGFR‐TKI. In this multicenter retrospective study, we collected data from mRCC pts receiving CN or NC, after frontline VEGFR‐TKI. A propensity score (PrS) was calculated to manage bias selection, and sequence comparisons were carried out with a cox model on a matched sample 1:1. The primary endpoint was progression‐free survival (PFS) from the start of second line to progression in third line (PFS_2‐3_). Key secondary endpoints included overall survival from second line (OS_2_). Out of 139 included mRCC patients, 38 (27%) and 101 (73%) received CN and NC, respectively. Overlap in PrS allowed 1:1 matching for each CN pts, with characteristics well balanced. For both PFS_2‐3_ and OS_2_, NC sequence was superior to CN (PFS_2‐3_: HR = 0.58 [0.34‐0.98], *P* = .043; OS_2_: 0.66 [0.42‐1.05], *P* = .080). Superior PFS_2‐3_ was in patients treated between 6 and 18 months with prior VEGFR‐TKI (*P* = .019) and was driven by a higher PFS_L3_ with cabozantinib when given after nivolumab (*P* < .001). The CABIR study shows a prolonged PFS of the NC sequence compared to CN in mRCC after first line VEGFR‐TKI failure. The data suggest that cabozantinib may be more effective than nivolumab in the third‐line setting, possibly related to an ability of cabozantinib to overcome resistance to PD‐1 blockade.

## What's new?

Cabozantinib and nivolumab both improve overall survival in patients with metastatic clear cell renal cell carcinoma previously treated with VEGFR‐TKI. This multicenter retrospective study is the first to compare the cabozantinib‐nivolumab and nivolumab‐cabozantinib sequences after failure of first line VEGFR‐TKI. The results of this matching‐adjusted comparison of 139 patients shows a superiority of nivolumab‐cabozantinib over cabozantinib‐nivolumab in progression‐free survival. The sequence's superiority, which was related to a higher efficacy of cabozantinib when administered in third line after nivolumab, suggests an ability of cabozantinib to overcome resistance to PD‐1 blockade.

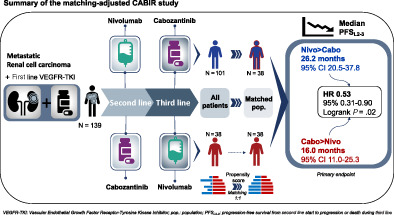

**Abbreviations**
CN: cabozantinib‐nivolumab sequence
CR: complete response
HR: hazard ratio
ICI: immune checkpoint inhibitor
IMDC: International Metastatic RCC Database Consortium
IQR: interquartile range
NC: nivolumab‐cabozantinib sequence
ORR: objective response rate
(m)OS: (median) overall survival
PD: progressive disease
PD‐1: programmed death‐1
PFS: progression‐free survival
PFS_2‐3_
: progression‐free survival of line 2 and 3
PFS_L2_
: progression‐free survival of line 2
PFS_L3_
: progression‐free survival of line 3
PR: partial response
PrS: propensity score
PS (or ECOG‐PS): performance status
(m)RCC: (metastatic) clear cell renal cell carcinoma
RECIST: Response Evaluation Criteria In Solid Tumors
SD: stable disease
VEGFR‐TKI: vascular endothelial growth factor receptor tyrosine kinase inhibitor

## 1 INTRODUCTION

The treatment landscape of metastatic clear cell renal cell carcinoma (mRCC) has flourished in the past few years and is continuously evolving.^1^ Currently, systemic therapies for mRCC focus on immune checkpoint inhibitor (ICI), vascular endothelial growth factor receptor tyrosine kinase inhibitor (VEGFR‐TKI) and more recently combination of both.^2^, ^3^ Nivolumab and cabozantinib are among the most frequently used therapies. Nivolumab was the first in class available ICI, targeting programmed death 1 (PD‐1) checkpoint.^4^ It was approved after the ChecKMate 025 randomized phase III study comparing nivolumab and everolimus in the second‐ and third line setting^5^ and is now widely used in monotherapy or in combination.^3^ Cabozantinib is a VEGFR‐TKI also targeting cMET and AXL.^6^ It was approved based on results from the METEOR randomized phase III study comparing cabozantinib and everolimus in the second‐ and third‐line setting^7^ and is now a major compound used in many settings of the mRCC management.^3^ Both nivolumab and cabozantinib monotherapies were approved at the same period and in the same setting, that is, after failure of a first‐line VEGFR‐TKI. In the absence of head‐to‐head comparison between these two drugs, clinicians cannot base their choice on evidence‐based data. The final results of the METEOR randomized phase III trial, showed cabozantinib efficacy in many subgroups, including in patients who had previously received nivolumab in second line.^8^, ^9^ In this small subgroup of 32 patients, superiority of cabozantinib over everolimus in median overall survival (mOS) and progression‐free survival (mPFS) were even higher than in the overall population with an HR 0.56 (95% CI, 0.21‐0.52) vs HR 0.66 (95% CI, 0.53‐0.83) and HR 0.22 (95% CI, 0.07‐0.65) vs HR 0.51 (95% CI, 0.41‐0.62), respectively. In contrast, cabozantinib superiority was similar after one or two VEGFR‐TKI, HR for PFS were 0.52 vs 0.51, respectively.^8^, ^9^ These results suggest that cabozantinib may be more effective when administered after nivolumab treatment. Another subgroup analysis from the METEOR trial showed that the PFS improvement with cabozantinib over everolimus was more pronounced in patients who had received >6 months of prior VEGFR‐TKI compared to ≤6 months, HR 0.48 vs 0.62, respectively.^9^

Thus, giving nivolumab followed by cabozantinib (NC) could be more effective than giving the reverse sequence (CN) in mRCC patients previously treated with one prior VEGFR‐TKI. Here, we investigated efficacy of both sequences as well as the potential predictive role of first‐line treatment duration on the subsequent lines.

## 2 MATERIAL AND METHODS

### 2.1 Patients

Patient data were collected retrospectively from 15 cancer centers in France. The main inclusion criterium was a clear cell metastatic renal cell carcinoma treated with either cabozantinib then nivolumab (CN) or nivolumab then cabozantinib (NC) in second‐ and third line, after failure of a first‐line VEGFR‐TKI (sunitinib or pazopanib). Patients should have had at least 6 months follow‐up in third line (or died during the third line). Patients with critical missing data for the propensity score model were excluded.

### 2.2 Outcomes

The primary endpoint was progression‐free survival of the overall sequence (line 2 and 3) defined as the time from the beginning of the second line of treatment to progression during third line or death (PFS_2‐3_). The secondary endpoint was overall survival from second line defined as the time from the beginning of the second line treatment to death (OS_2_). Exploratory endpoints were progression‐free survival during second (PFS_L2_) and third lines (PFS_L3_), and objective response (RECIST 1.1) during second‐ and third lines.

### 2.3 Statistical analysis

We planned two main analyses, one for the sequence effect and one for the first line duration effect. For comparison of the two sequences, a propensity score (PrS) was calculated to reduce bias selection. As the number of patients was small, variable selection was based on clinician's opinion. We selected five variables clinically relevant for the choice of the second‐line treatment and related to the primary endpoint (PFS_2‐3_): first‐line VEGFR‐TKI treatment duration, best response (RECIST 1.1), reason for discontinuation, bone metastasis before first line (yes/no) and bone metastasis before second line (yes/no). These five variables were included in a logistic regression model to calculate PrS for each patient (sequence VEGFR‐TKI‐CN was set as reference). A one‐to‐one (1:1) matching without replacement was then performed to create a matched population.^10^, ^11^, ^12^, ^13^ Hazard ratios (HR) from cox models and Kaplan‐Meier curves were estimated in the matched population to assess sequence effectiveness through second‐ and third lines. For the analysis of first line duration effect, cox models and Kaplan‐Meier curves were used in the overall population, for each sequence separately. For all the analyses, duration of first‐line treatment was transformed into a three‐class categorical variable with cut‐off at 6 and 18 months as it was considered clinically meaningful and robust to outliers. Sensibility analyses for the sequence effect were performed on a weighted 1:2 matched population (where patients of the CN sequence were taken twice to double their weights and make a 76 vs 76 comparisons groups) and overall population (with PrS as adjusting covariate). We used R (https://www.r-project.org) and package “Matching”^12^ for all the analysis.

## 3 RESULTS

### 3.1 Patients

Between March 2016 and August 2020, 139 eligible patients were enrolled, 38 (27%) received the CN sequence and 101 (73%) received the NC sequence. Median follow‐up (from the beginning of the second line) were 19.7 and 24.1 months for CN and NC, respectively. Patients' characteristics were slightly unbalanced in the overall population but well‐balanced in the matched population (Table 1). Overlap of PrS distribution in both sequences was enough to match each patient in the CN sequence with one or two patients in the NC sequence (Figures S1 and S2).

**TABLE 1:** Patients' characteristics

	Overall population			Matched (1:1) population		
Variable	Cabo‐Nivo, N = 38	Nivo‐Cabo, N = 101	*P*‐value^a^	Cabo‐Nivo, N = 38	Nivo‐Cabo, N = 38	*P*‐value^a^
Age			.39			.36
Median [IQR], years	65 [61, 70]	67 [58, 73]		65 [61, 70]	67 [58, 74]	
Sex			.59			.50
Female	6/38 (16%)	20/101 (20%)		6/38 (16%)	4/38 (11%)	
Male	32/38 (84%)	81/101 (80%)		32/38 (84%)	34/38 (89%)	
Nephrectomy	26/34 (76%)	83/97 (86%)	.22	26/34 (76%)	31/37 (84%)	.44
Unknown	4	4		4	1	
First‐line treatment			.079			.19
Sunitinib	26/38 (68%)	83/101 (82%)		26/38 (68%)	31/38 (82%)	
Pazopanib	12/38 (32%)	18/101 (18%)		12/38 (32%)	7/38 (18%)	
First‐line duration			.24			.56
Median [IQR], mo	12 [8, 21]	10 [5, 21]		12 [8, 21]	13 [9, 25]	
First line duration by class			.53			.24
<6 mo	7/38 (18%)	28/101 (28%)		7/38 (18%)	2/38 (5.3%)	
6‐18 mo	18/38 (47%)	43/101 (43%)		18/38 (47%)	21/38 (55%)	
>18 mo	13/38 (34%)	30/101 (30%)		13/38 (34%)	15/38 (39%)	
Responder during first line	21/38 (55%)	36/101 (36%)	.036	21/38 (55%)	20/38 (53%)	.82
Reason for first line discontinuation			.057			>.99
Progression	35/38 (92%)	79/101 (78%)		35/38 (92%)	35/38 (92%)	
Toxicity	3/38 (7.9%)	22/101 (22%)		3/38 (7.9%)	3/38 (7.9%)	
Bone metastasis at first line start	19/38 (50%)	39/101 (39%)	.23	19/38 (50%)	18/38 (47%)	.82
Bone metastasis at second line start	23/38 (61%)	47/101 (47%)	.14	23/38 (61%)	24/38 (63%)	.81
ECOG PS at second line start			.70			.84
0	11/38 (29%)	30/101 (30%)		11/38 (29%)	9/38 (24%)	
1	19/38 (50%)	56/101 (55%)		19/38 (50%)	23/38 (61%)	
2	7/38 (18%)	14/101 (14%)		7/38 (18%)	5/38 (13%)	
3	1/38 (2.6%)	1/101 (1.0%)		1/38 (2.6%)	1/38 (2.6%)	
IMDC at second line start			.56			>.99
Good	3/38 (7.9%)	13/101 (13%)		3/38 (7.9%)	3/38 (7.9%)	
Inter/poor	35/38 (92%)	88/101 (87%)		35/38 (92%)	35/38 (92%)	
Number of metastasic sites at second line start			.51			>.99
1	2/38 (5.3%)	13/101 (13%)		2/38 (5.3%)	3/38 (7.9%)	
2	13/38 (34%)	33/101 (33%)		13/38 (34%)	13/38 (34%)	
3+	23/38 (61%)	55/101 (54%)		23/38 (61%)	22/38 (58%)	

*Note*: Data are n (%) or median [IQR]. Main clinical characteristics of all patients (overall population, n = 139) included in the study and in matched (1:1) population of patients (n = 38).

Abbreviations: CN, cabozantinib‐nivolumab sequence; ECOG PS, eastern cooperative oncology group performance status; F, female; IMDC, international metastatic RCC database consortium; IQR, interquartile range; M, male; NC, nivolumab‐cabozantinib sequence; PD, progressive disease; Tox, toxicities.

^a^ Wilcoxon rank sum test; Pearson's chi‐squared test; Fisher's exact test.

### 3.2 Outcomes according to treatment sequence

**FIGURE 1:**
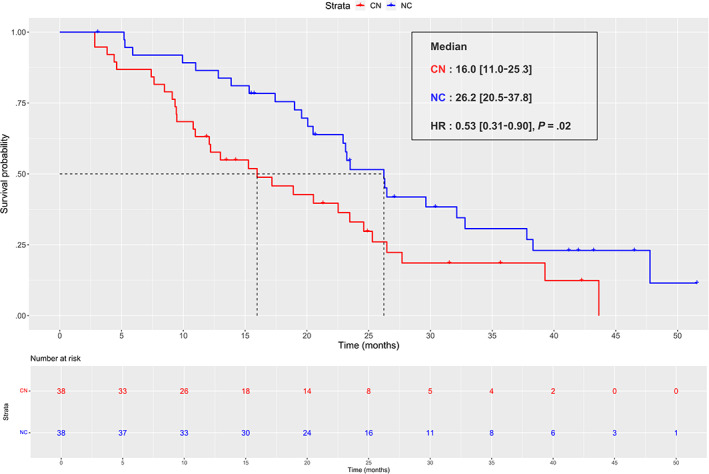
Progression‐free survival from L2 to L3 (PFS2‐3) in matched (1:1) population. Kaplan‐Meier curve of progression‐free‐survival from second line to third line in matched (1:1) population (n = 38). The red curve represents the cabozantinib‐nivolumab (CN) sequence and the blue curve represents the nivolumab‐cabozantinib (NC) sequence [Color figure can be viewed at wileyonlinelibrary.com]

In the matched population, median PFS_2‐3_ were 26.2 (95% CI, 20.5‐37.8) and 16.0 (95% CI, 11.0‐25.3) months for the NC and CN sequences, respectively. Superiority of NC was significant (HR = 0.53 [95% CI, 0.31‐0.90], *P* = .02) (Figure 1). For OS_2_ median were 32.9 and 32.0 months for the NC and CN, respectively (Figure S3), with no difference (HR = 0.81 [95% CI, 0.43‐1.53], *P* = .52).

**FIGURE 2:**
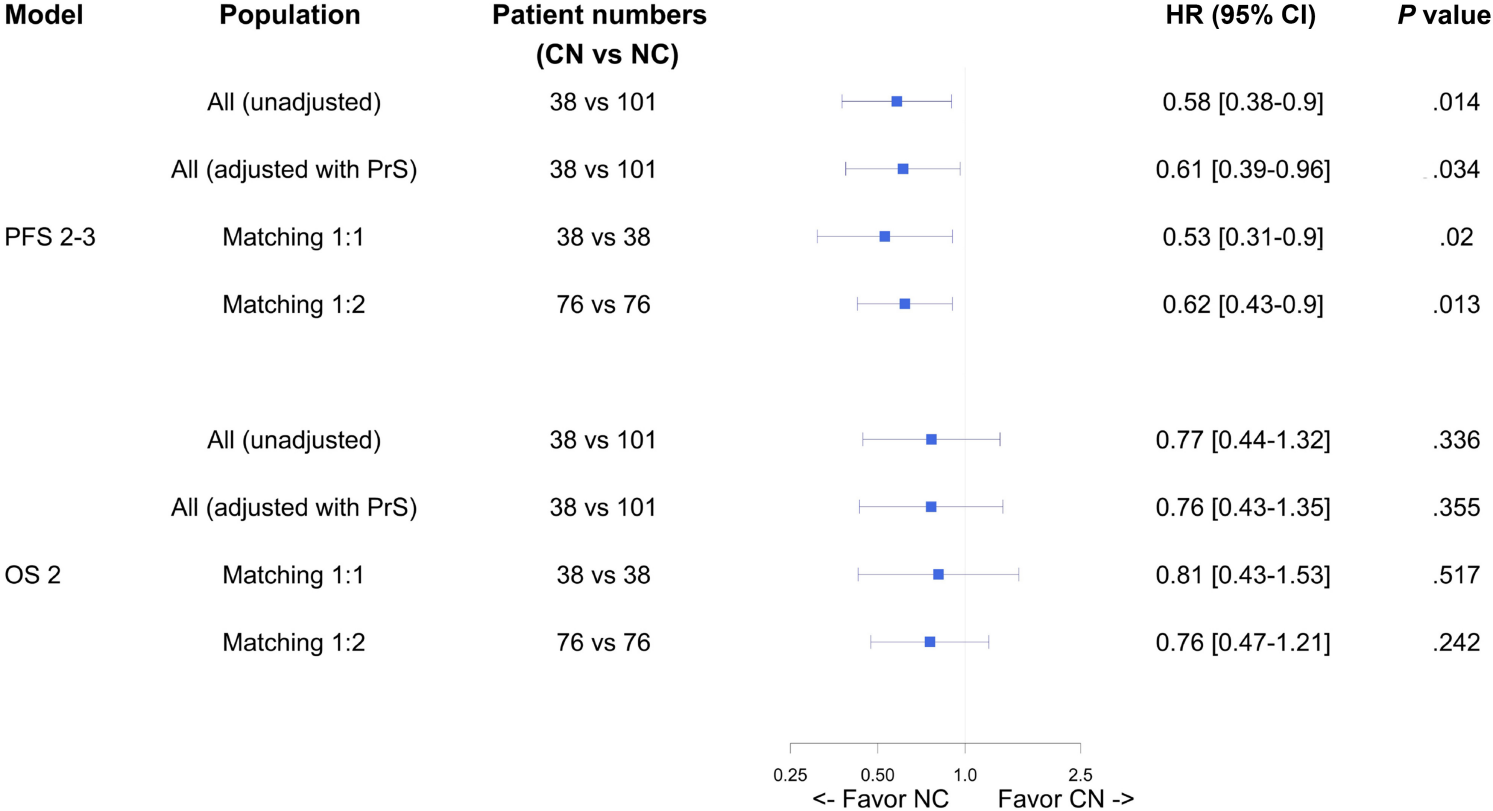
Forest plot of sequence effect (PFS2‐3 and OS2) in different models. Forest plot representing the effect of cabozantinib‐nivolumab (CN) and nivolumab‐cabozantinib (NC) sequences on progression‐free‐survival from line 2 to line 3 (PFS_2‐3_) and on overall survival from line 2 (OS_2_), according to the following models: all patients (n = 139), all patient with adjustment on propensity score (PrS, n = 139), 1:1 matched population (n = 38), 1:2 matched population (n = 76). Hazard ratio below 1 favors the NC sequence [Color figure can be viewed at wileyonlinelibrary.com]

In the overall population (with PrS as adjusting covariate) and weighted 1:2 matched population, we found similar results: the NC sequence was again superior over CN (overall population: HR = 0.61 [95% CI, 0.39‐0.96]; matched 1:2 population: HR = 0.62 [95% CI, 0.43‐0.90]), and no difference was found for OS_2_ (Figure 2).

### 3.3 Outcomes according to treatment line

**FIGURE 3:**
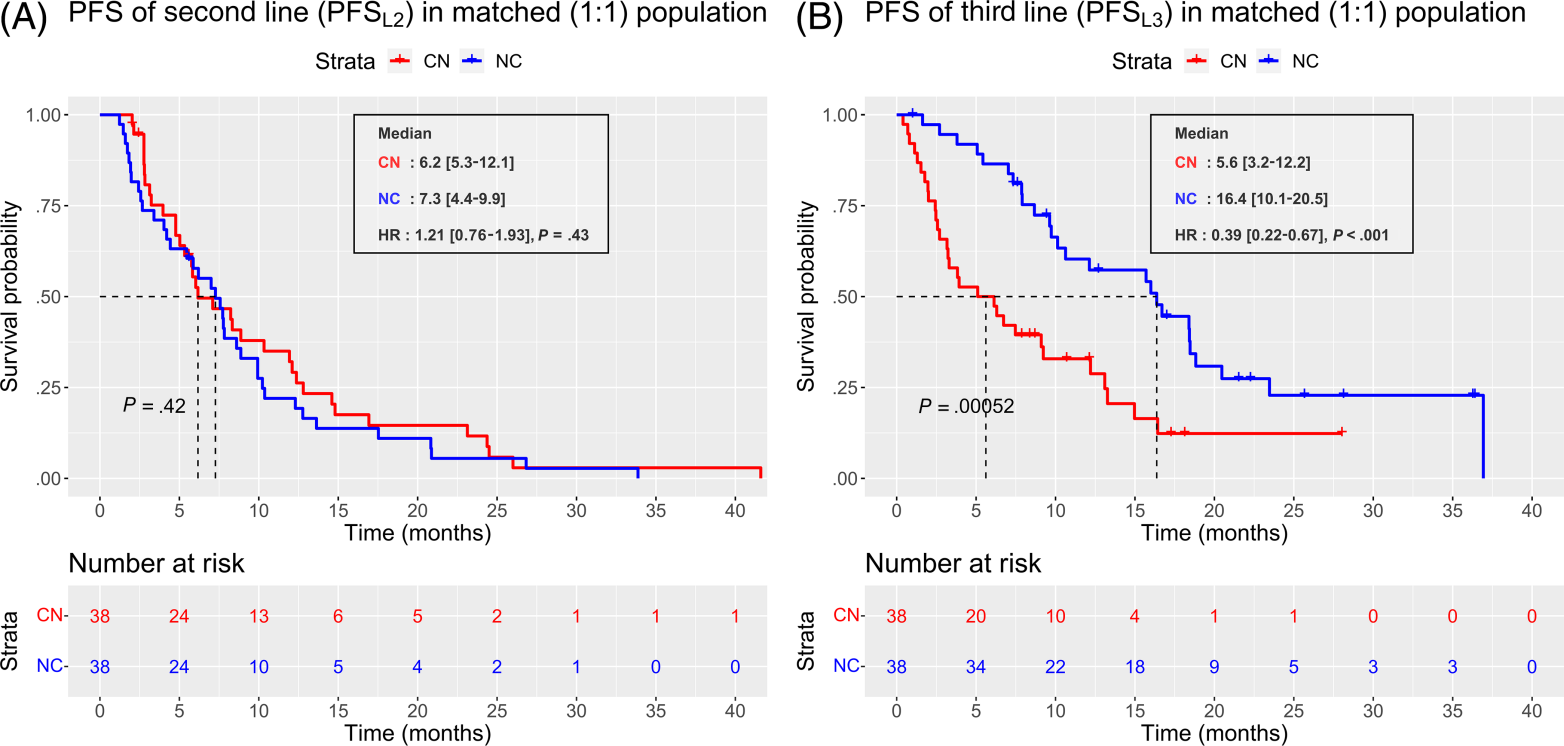
Progression‐free survival (PFS) in second and third lines. Kaplan‐Meier curves of progression‐free‐survival (PFS) in second (A) and third line (B). The red curve represents the cabozantinib‐nivolumab (CN) sequence and the blue curve represents the nivolumab‐cabozantinib (NC) sequence [Color figure can be viewed at wileyonlinelibrary.com]

**FIGURE 4:**
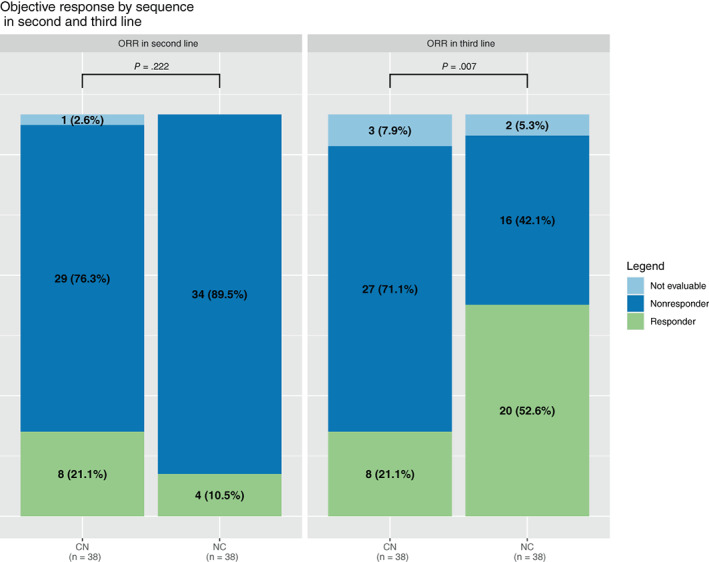
Objective response rate (ORR) during second and third lines. Histogram representing the objective response rate (RECIST 1.1) with cabozantinib and nivolumab in second (left) and third line (right) in matched (1:1) population (n = 38). Green bars represent responders (complete and partial), dark blue bars represent nonresponders (progression and stable disease) and light blue bars represent nonevaluable patients. CN, cabozantinib‐nivolumab; NC, nivolumab‐cabozantinib [Color figure can be viewed at wileyonlinelibrary.com]

When considering second‐ and third line separately, there was little to no difference between nivolumab and cabozantinib efficacy in second line (PFS_L2_: HR = 1.21 [95% CI, 0.76‐1.93], *P* = .43). In contrast, cabozantinib efficacy in third line was higher when given after nivolumab (NC sequence), HR = 0.39 [95% CI, 0.22‐0.67], *P* < .001 (Figure 3). Similar results were obtained regarding the objective response rate, with a slight advantage for cabozantinib over nivolumab in second line (21% vs 11%), and a large advantage of cabozantinib over nivolumab in third line (53% vs 21%), respectively (Figure 4). Interestingly, patients' characteristics remained comparable between the two sequences at the beginning of the third line (Table S1).

### 3.4 Outcomes according to first line duration

**FIGURE 5:**
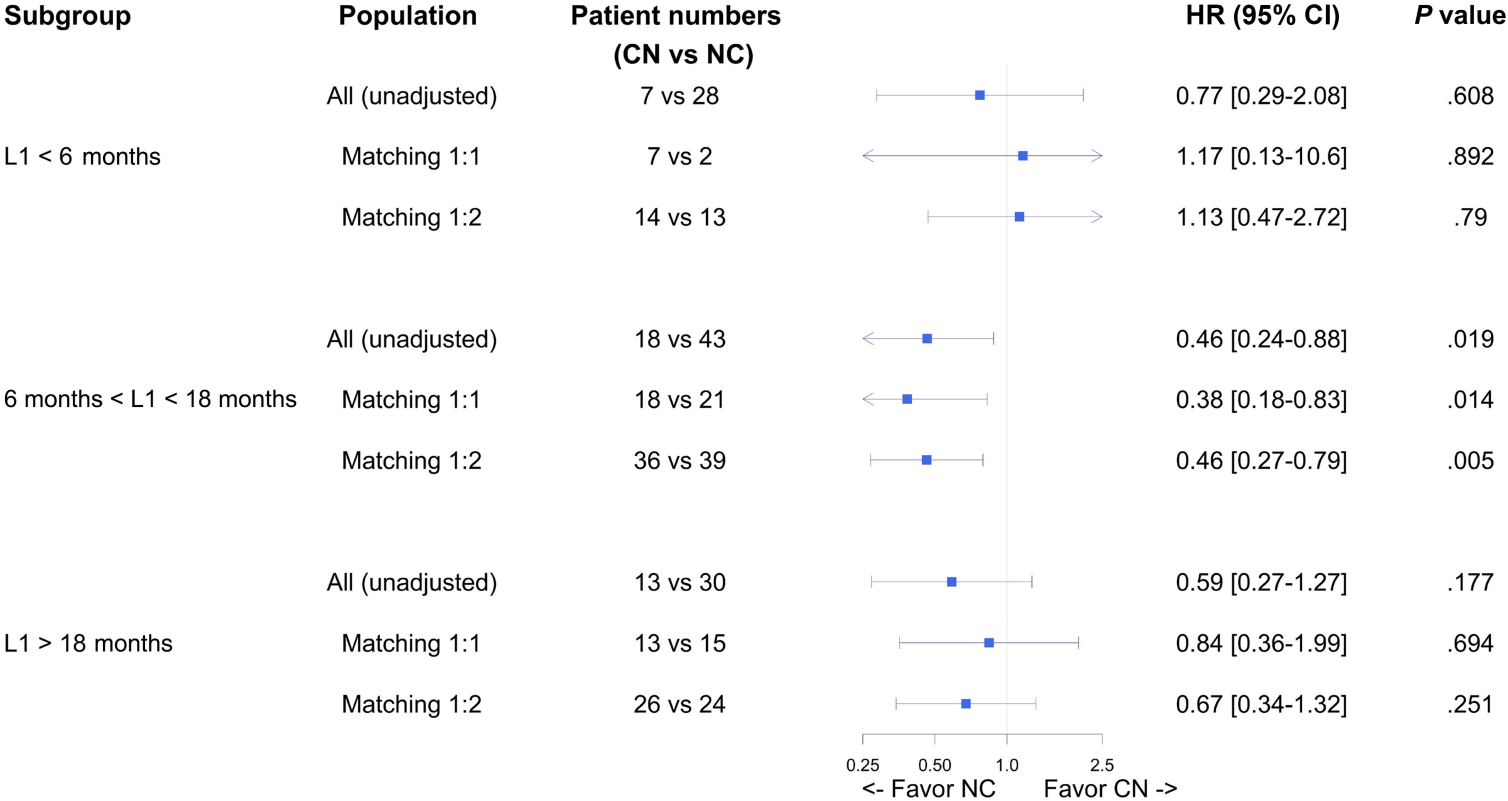
Forest plot of sequence effect (PFS2‐3) by subgroups. Forest plot representing the effect of cabozantinib‐nivolumab (CN) and nivolumab‐cabozantinib (NC) sequences on progression‐free‐survival (PFS) from line 2 to line 3, according to frontline VEGFR‐TKI duration (<6 months, 6‐18 months and >18 months) in the following models: all patients, 1:1 matched population, 1:2 matched population. Hazard ratio below 1 favors the NC sequence [Color figure can be viewed at wileyonlinelibrary.com]

Analysis by duration of first‐line therapy within three classes (<6 months, 6‐18 months, >18 months) showed a difference in PFS between sequences only for patients treated between 6 and 18 months (Figure 5). No difference in mOS was found between sequences according to the three classes of first‐line treatment duration (Figure S4). We then assessed each sequence effectiveness followed by each treatment separately used in second‐ and third line according to the first‐line treatment duration.

For the overall CN sequence, first‐line treatment duration had no effect on mPFS_2‐3_ (*P* = .5). Cabozantinib used in second line showed improved mPFS (*P* = .044) and ORR (*P* = .005) for patients with first line VEGFR‐TKI duration >18 months whereas nivolumab efficacy in third line was not influenced by first line VEGFR‐TKI duration (Table S2).

For the NC sequence, first‐line treatment duration had no effect on mPFS_2‐3_ (*P* = .2). Nivolumab used in second line as well as cabozantinib used in third line showed similar efficacy across all first line VEGFR‐TKI duration subgroups (Table S3). Of note, cabozantinib had a notable high efficacy across the three subgroups with mPFS_L3_ of 12.9, 11.1 and 16.0 months (*P* = .4) and ORR of 39%, 47% and 50% (*P* = .46) in patients treated with VEGFR‐TKI in first line <6 months, between 6 and 18 months and >18 months, respectively (Table S3).

## 4 DISCUSSION

In this multicenter retrospective study, we compared CN vs NC sequences after one prior VEGFR‐TKI in patients with mRCC matched on PrS and investigated the effect of first line duration of VEGFR‐TKI on subsequent lines efficacy. We showed that the NC sequence leads to better outcomes compared to CN, with higher PFS and objective response rate, particularly in patients treated between 6 and 18 months with first line sunitinib or pazopanib. We further showed that this sequence superiority could be explained by a higher efficacy of cabozantinib in third line after nivolumab.

No randomized prospective study has ever compared NC vs CN sequences. Our study differed from other retrospective or observational studies by two major points: first, we predefined two precise sequences while other studies had broader settings, mainly grouping different lines together, which made the comparison difficult. Second, we used a propensity score to manage bias selection, with five clinically relevant variables for the choice of second‐line treatment. Due to a small number of patients available in the CN sequence, performing a statistical variable selection or adding too many variables in the PrS model would have led to difficulties to balance population's characteristics. Therefore, we based our variable selection upon clinician's opinions. Specifically, bone metastasis before first line and second line were both in the model as we noticed some patients having bone metastasis in first line but not in second line, and that could have influenced practician's choice in a real‐world patient's follow‐up scenario. Moreover, variables such as ECOG‐PS or IMDC were not selected to be put in the PrS model as they mainly influence OS and not PFS and overall, these variables were well balanced in the matched population. While these statistical methods are not as powerful as a randomized trial, they provided a more robust and reliable comparison of efficacy of these two sequences than other nonrandomized trials.

To our knowledge, we report the highest response rate (53%) and longest PFS (16.4 months) with cabozantinib or with any other VEGFR‐TKI monotherapy in third line setting compared to previously published studies.^6^, ^7^, ^8^ In the METEOR trial, Powles et al^9^ reported a greater PFS superiority of cabozantinib over everolimus in the ICI pretreated subgroup (HR = 0.22) than the non‐pre‐treated one (HR = 0.54). Shah et al reported comparable efficacy in 20 patients treated with cabozantinib post‐ICI with an ORR of 45% and a median PFS of 15.2 months.^14^ Similarly, McGregor et al reported an ORR of 42% in 55 patients treated with cabozantinib after ICI or ICI‐ICI therapies, with no difference when prior ICI was given in first or ≥second line (43% vs 41%, respectively).^15^ In line with our observations, preliminary results from the real‐world CASSIOPE observational study of post‐VEGFR‐TKI, cabozantinib reported an 40% ORR when given in third line after nivolumab vs 24.5% when given in second, and 36% in third line, respectively.^16^ Consistently, in the French real‐world observational study CABOREAL, Albiges et al showed that prior treatment with nivolumab prolonged the time to treatment discontinuation with cabozantinib (*P* = .0008).^17^ The BREAKPOINT study, a prospective single arm phase II trial, reported an ORR of 43% and mPFS of 9.3 months in 49 patients treated in second line with cabozantinib after ICI‐based therapy.^18^

Altogether, these results and ours show that cabozantinib may have a better efficacy after nivolumab than before. It is not yet known whether this higher activity is specifically related to cabozantinib or whether it applies more broadly to the entire class of VEGFR‐TKIs. Marteau et al reported significantly higher ORR and longer time to treatment discontinuation with cabozantinib compared to other VEGR‐TKI after ICI therapy in a large cohort of 247 patients, with 54% vs 38% (*P* = .0002) and 6.2 months vs 3.1 months (*P* = .0052), respectively.^19^ Similarly in a post‐ICI setting, Santini et al reported longer PFS with cabozantinib compared with everolimus and other VEGFR‐TKI, 7.6 vs 3.2 vs 4.3 months, respectively (HR: 0.2 [95% CI, 0.1026‐0.7968] and HR: 0.6 [95% CI, 0.35‐1.23], respectively).^20^ These results argue for a specific efficacy of cabozantinib after anti‐PD‐1, the underlying mechanisms of which are not fully understood. However, it has been showed that AXL, one of the main targets of cabozantinib, promotes tumor immune evasion and is a candidate factor of resistance to PD‐1 blockade.^21^, ^22^

First‐line treatment duration affected sequence effectiveness for patients treated for 6 to 18 months, which was the largest subgroup of patients in our study. No difference was found between the two sequences when patients were treated less than 6 months or over 18 months. Hypothetically, patients treated less than 6 months may have a refractory and fast‐growing disease with no effective treatment, whereas those treated more than 18 months may have a more indolent and slow‐growing disease with a potential to respond to many treatment types. Regarding patients treated between 6 and 18 months, the difference between NC and CN was even more compelling than in the overall cohort. This finding is in line with previous studies reporting an association between cabozantinib's efficacy and the duration of first‐line VEGFR‐TKI. In the METEOR trial, mPFS and ORR with cabozantinib were higher in patients with ≥6 months of prior VEGFR‐TKI than in patients <6 months, 9 vs 5.6 months and 19 vs 14%, respectively.^9^ Interestingly, we report the same observation that patients receiving cabozantinib as second‐line therapy had longer mPFS when their first‐line VEGFR‐TKI was prolonged (>6 months); in contrast, patients receiving cabozantinib in the third line after nivolumab have a high PFS that is not influenced by the duration of the first‐line VEGFR‐TKI. This observation adds further support to a possible ability of cabozantinib to reverse resistance to PD‐1 blockade, as described above. Thus, targeting AXL after PD‐1 blockade failure may be an effective strategy supported by biological and clinical data.

We found no difference in overall survival between sequences, which may be explained by several factors. Some potentially related to the limitations of our study are described below. However, the main and unanimously recognized prognostic factor for survival in mRCC from first to third line is the IMDC risk group.^3^ IMDC is used as a stratification factor in all pivotal phase III randomized trials. In our matched population, the IMDC groups are perfectly balanced between the two sequences. Thus, it is not surprising that patients from both sequences have a comparable survival. Therefore, we believe that our results regarding the superior PFS of one sequence over the other do not reflect any confounding prognostic factor. In the absence of robust biomarkers that would be necessary to allow the selection of the most appropriate sequence for each patient, our results may be used to help the clinician in his or her therapeutic decision.

This retrospective study presents several limitations. First, the sample size of the study is limited; in addition, matching led to further reduce the sample size once we matched patients in both sequences. Second, we had a lot more patients with NC than CN sequence because in France, nivolumab was reimbursed before cabozantinib in the same setting. Thus, more patients are needed in the CN sequence to be more representative of the real‐world population. Even though HRs did not seem to vary a lot with the sensibility analysis on the overall population (139 patients), estimations should be taken with caution, considering their confidence intervals. Third, even though we tried to balance patients' characteristics, we selected patients with at least three treatments lines and two specific sequences (CN or NC), excluding other treatments. It is likely that this selection increased patients' survival estimations in both sequences. Fourth, this study was conducted using data collected only in French centers. Clinicians' practices may slightly differ from one country to another. Fifth, as the VEGR‐TKI monotherapy has been replaced by ICI‐based combination in frontline treatment of mRCC,^3^ we do not know whether our results could be applicable with cabozantinib in second line after frontline of ICI based combinations. This question will be addressed by the CaboPoint (NCT03945773) single arm phase II study evaluating the efficacy and safety of cabozantinib in second‐line setting after ICI‐based combination, which is currently recruiting. Finally, the reduced use of a VEGFR‐TKI in the first line of treatment may reduce the significance of our results. Nevertheless, many patients are currently being treated with a TKI in first line and will be candidates for a second line in the coming months. In addition, a number of patients seen on a routine basis are more frail than those included in clinical trials and may not be good candidates for combination therapy (a decision that depends on the prescriber's experience but also on the availability of the molecules), including but not limited to: patients in poor general condition, very old and frail patients; patients with severe comorbidities; and patients on long‐term corticosteroid therapy. Finally, for patients in the favorable IMDC arm, the updated Keynote 426 study did not show a PFS (HR 0.76 [95% CI 0.56‐1.03]) or OS (HR 1.17 [95% CI 0.76‐1.80]) benefit of pembrolizumab‐axitinib over sunitinib^23^; in our opinion, VEGFR‐TKI monotherapy remains a valid option in these patients.

## 5 CONCLUSION

The current matching‐adjusted study shows a prolonged PFS with nivolumab‐cabozantinib compared with the cabozantinib‐nivolumab sequence in patients with mRCC. This superiority, particularly in patients who received 6 to 18 months of first‐line VEGFR‐TKI, may be due to the greater efficacy of cabozantinib when administered in the third line after nivolumab. These results support the hypothesis that the AXL pathway, one of the targets of cabozantinib, may be an escape route to PD‐1 blockade and that cabozantinib may overcome this resistance.

## AUTHOR CONTRIBUTIONS

*Conceptualization*: Yann‐Alexandre Vano, Réza‐Thierry Elaidi, Stéphane Oudard. *Data curation*: Letuan Phan. *Formal analysis*: Letuan Phan. *Funding acquisition*: Yann‐Alexandre Vano, Réza‐Thierry Elaidi, Stéphane Oudard. *Data collection (enrollment)*: Yann‐Alexandre Vano, Gwenaelle Gravis, Iphigénie Korakis, Friederike Schlürmann, Denis Maillet, Mostefa Bennamoun, Nadine Houede, Delphine Topart, Delphine Borchiellini, Philippe Barthelemy, Raffaele Ratta, Thomas Ryckewaert, Ali Hasbini, Sophie Hans, Sheik Emambux, Stéphane Oudard. *Methodology*: Réza‐Thierry Elaidi, Yann‐Alexandre Vano, Stéphane Oudard. *Project administration*: EB and SC (administrative coordination), Yann‐Alexandre Vano (scientific and clinical coordination). *Supervision*: Yann‐Alexandre Vano, Letuan Phan, Stéphane Oudard. *Visualization*: Letuan Phan. *Writing—original draft*: Letuan Phan, Yann‐Alexandre Vano. *Writing—review & editing*: all coauthors. The work reported in the paper has been performed by the authors, unless clearly specified in the text.

## FUNDING INFORMATION

IPSEN Pharma. The funder's role was limited to providing financial support for the organization of the data collection. The funder had no direct role in data collection, analysis and writing the manuscript.

## CONFLICT OF INTEREST

Yann‐Alexandre Vano received consultancy fees from BMS, MSD, Ipsen, Merck, Pfizer, Novartis. Gwenaelle Gravis: consultancy fees were received by her institution from BMS, Pfizer, MSD, Alliance Merck‐Pfizer; fees received by her institution for coordinating PI role from BMS. Denis Maillet received consultancy fees from BMS, Ipsen, Pfizer, MSD. Delphine Borchiellini: received consultancy fees from Astellas, AstraZeneca, Bayer, BMS, Ipsen, Janssen, Merck, MSD, Pfizer; clinical research funding (institution) from: Astellas, AstraZeneca, Bayer, BMS, Exelixis, Infinity, Janssen, MSD, Pfizer, Roche, Taiho Oncology. Philippe Barthelemy received consultancy fees from BMS, Ipsen Pfizer, Merck MSD Novartis. Raffaele Ratta received consultancy fees from BMS, MSD, Ipsen, Merck, Pfizer. Sheik Emambux received consultancy fees from BMS and IPSEN. Stéphane Oudard received consultancy fees from Merck, Novartis, Pfizer, BMS, Ipsen. Mostefa Bennamoun, Letuan Phan, Iphigénie Korakis, Friederike Schlürmann, Nadine Houede, Delphine Topart, Thomas Ryckewaert, Ali Hasbini, Sophie Hans, Sandra Cournier, Elena Braychenko, Réza‐Thierry Elaidi: none.

## ETHICS STATEMENT

The study was conducted in accordance with the principles of Good Clinical Practice and the Declaration of Helsinki, and was approved by an independent ethics committee. All included patients gave their informed consent.

## Supporting information


**Table S1** Patients' characteristics at third line (matched population)
**Table S2** Efficacy of cabozantinib‐nivolumab sequence according to duration of VEGFR‐TKI in first line
**Table S3** Efficacy of nivolumab‐cabozantinib sequence according to duration of VEGFR‐TKI in first line
**Figure S1** Propensity score distribution in overall population
**Figure S2** Propensity score distribution in matched population
**Figure S3** Overall survival in second line (OS2) in matched 1:1 population
**Figure S4** Overall survival in second line (OS2) by first line duration subgroups in matched 1:1 population

## Data Availability

Individual data will be available only upon proposal from investigators and following approval by an independent review committee (“learned intermediary”) identified for this purpose. Proposals should be directed to yann.vano@aphp.fr. To gain access, data requestors will need to sign a data access agreement. Further information is available from the corresponding author upon request.
