# Supplementary material for: Cabozantinib‐nivolumab sequence in metastatic renal cell carcinoma: The CABIR study
Source: Int J Cancer. 2022 Jun 6;151(8):1335–44. doi: 10.1002/ijc.34126 (PMC9541795; doi:10.1002/ijc.34126)
Supplement: Supplementary file 1 — Table S1 Patients' characteristics at third line (matched population) Table S2 Efficacy of cabozantinib‐nivolumab sequence according to duration of VEGFR‐TKI in first line Table S3 Efficacy of nivolumab‐cabozantinib sequence according to duration of VEGFR‐TKI in first line Figure S1 Propensity score distribution in overall population Figure S2 Propensity score distribution in matched population Figure S3 Overall survival in second line (OS2) in matched 1:1 population Figure S4 Overall survival in second line (OS2) by first line duration subgroups in matched 1:1 population [file IJC-151-1335-s001.pdf]

Supplementary Material

Cabozantinib-nivolumab sequence in metastatic renal cell carcinoma: the CABIR study

Yann-Alexandre Vano, MD; Letuan Phan, PhD; Gwenaelle Gravis, MD; Iphigénie Korakis, MD; Friederike Schlürmann, MD; Denis Maillet, MD; Mostefa Bennamoun, MD; Nadine Houede, MD; Delphine Topart, MD; Delphine Borchiellini, MD; Philippe Barthelemy, MD; Raffaele Ratta, MD; Thomas Ryckewaert, MD; Ali Hasbini, MD; Sophie Hans, MD; Sheik Emambux, MD; Sandra Cournier; Elena Braychenko; Réza-Thierry Elaidi, PhD; Stéphane Oudard, MD.

Table of contents

SUPPLEMENTARY TABLES **2**

TABLE S1 – PATIENTS' CHARACTERISTICS AT 3RD LINE (MATCHED POPULATION) 2

TABLE S2 – EFFICACY OF CABOZANTINIB-NIVOLUMAB SEQUENCE ACCORDING TO DURATION OF VEGFR-TKI IN 1ST LINE 3

TABLE S3 – EFFICACY OF NIVOLUMAB-CABOZANTINIB SEQUENCE ACCORDING TO DURATION OF VEGFR-TKI IN 1ST LINE 4

SUPPLEMENTARY FIGURES **5**

FIGURE S1 – PROPENSITY SCORE DISTRIBUTION IN OVERALL POPULATION 5

FIGURE S2 – PROPENSITY SCORE DISTRIBUTION IN MATCHED POPULATION 6

FIGURE S3 –OVERALL SURVIVAL IN 2ND LINE (OS_2) IN MATCHED 1:1 POPULATION 7

FIGURE S4 –OVERALL SURVIVAL IN 2ND LINE (OS_2) BY 1ST LINE DURATION SUBGROUPS IN MATCHED 1:1 POPULATION 8

Supplementary tables

Table S1 – Patients' characteristics at 3rd line (matched population)

Variable	N	CN, N = 38	NC, N = 38	p-value[1]
**Duration of 2nd line**	76			0.29
Median [IQR]		7 [3, 12]	6 [3, 10]	
**Responder during 2nd line**	76			0.22
Yes		8 / 38 (21%)	4 / 38 (11%)	
No		29 / 38 (76%)	34 / 38 (89%)	
Not evaluable		1 / 38 (2.6%)	0 / 38 (0%)	
**Reason for 2nd line discontinuation**	76			0.67
Investigator's choice		0 / 38 (0%)	1 / 38 (2.6%)	
Progression		34 / 38 (89%)	35 / 38 (92%)	
Toxicity		4 / 38 (11%)	2 / 38 (5.3%)	
**IMDC at 3rd line start**	76			0.57
Good		4 / 38 (11%)	2 / 38 (5.3%)	
Intermediate		19 / 38 (50%)	23 / 38 (61%)	
Poor		15 / 38 (39%)	13 / 38 (34%)	
**ECOG PS at 3rd line start**	73			0.93
0		6 / 36 (17%)	6 / 37 (16%)	
1		20 / 36 (56%)	18 / 37 (49%)	
2		8 / 36 (22%)	10 / 37 (27%)	
3		2 / 36 (5.6%)	3 / 37 (8.1%)	
Unknown		2	1	

[1]Wilcoxon rank sum test; Fisher's exact test

CN: cabozantinib-nivolumab sequence; NC: nivolumab-cabozantinib sequence; IMDC: international metastatic RCC database consortium; ECOG PS: eastern cooperative oncology group performance status; IQR: interquartile range.

Caption: Patients' characteristics in 1:1 matched population (n=38) before the beginning of the third line treatment.

Table S2 – Efficacy of cabozantinib-nivolumab sequence according to duration of VEGFR-TKI in 1st line

	Best response (RECIST 1.1)							Median Survival	
	Treatment	CR	PR	SD	PD	NE	p-value[1]	Median PFS, months (95% CI)	p-value[2]
**In 2nd line**	Cabo						0.005		0.044
VEGFR-TKI < 6 mo	Cabo	0 / 7 (0%)	1 / 7 (14%)	2 / 7 (29%)	4 / 7 (57%)	0 / 7 (0%)		4.0 (2.8 to NE)	
6 mo < VEGFR-TKI < 18 mo	Cabo	0 / 18 (0%)	0 / 18 (0%)	11 / 18 (61%)	6 / 18 (33%)	1 / 18 (5.6%)		7.1 (4.8 to 15)	
VEGFR-TKI > 18 mo	Cabo	0 / 13 (0%)	7 / 13 (54%)	4 / 13 (31%)	2 / 13 (15%)	0 / 13 (0%)		12 (6.0 to NE)	
**In 3rd line**	Nivo						0.76		0.13
VEGFR-TKI < 6 mo	Nivo	0 / 7 (0%)	2 / 7 (29%)	1 / 7 (14%)	4 / 7 (57%)	0 / 7 (0%)		5.1 (1.5 to NE)	
6 mo < VEGFR-TKI < 18 mo	Nivo	0 / 18 (0%)	3 / 18 (17%)	4 / 18 (22%)	10 / 18 (56%)	1 / 18 (5.6%)		3.5 (2.0 to 9.2)	
VEGFR-TKI > 18 mo	Nivo	1 / 13 (7.7%)	2 / 13 (15%)	4 / 13 (31%)	4 / 13 (31%)	2 / 13 (15%)		12 (3.3 to NE)	
**PFS $_{2\text{-}3}$**									0.5
VEGFR-TKI < 6 mo								11 (7.4 to NE)	
6 mo < VEGFR-TKI < 18 mo								14 (9.3 to NE)	
VEGFR-TKI > 18 mo								23 (11 to NE)	

[1]Fisher's exact test ; [2]Log-rank test

Cabo: cabozantinib; Nivo: nivolumab; VEGFR-TKI: vascular endothelial growth factor receptor tyrosine kinase inhibitor; CR: complete response; PR: partial response; SD: stable disease; PD: progressive disease; NE: not evaluable; mo: months; PFS$_{2\text{-}3}$: progression-free survival from 2nd line start to progression or death during 3rd line.

Caption: Best objective response and median progression-free survival (PFS) using RECIST 1.1 by investigator with cabozantinib received in second line, nivolumab received in third line, and cabozantinib-nivolumab sequence (PFS$_{2\text{-}3}$ only) according to duration of VEGFR-TKI treatment in first line.

Table S3 – Efficacy of nivolumab-cabozantinib sequence according to duration of VEGFR-TKI in 1st line

	Best response (RECIST 1.1)							Median Survival	
	Treatment	CR	PR	SD	PD	NE	p-value[1]	Median PFS (95% CI)	p-value[2]
**In 2nd line**	Nivo						0.13		0.9
VEGFR-TKI < 6 mo	Nivo	0 / 28 (0%)	1 / 28 (3.6%)	10 / 28 (36%)	16 / 28 (57%)	1 / 28 (3.6%)		4.8 (2.4 to 7.6)	
6 mo < VEGFR-TKI < 18 mo	Nivo	0 / 43 (0%)	7 / 43 (16%)	16 / 43 (37%)	20 / 43 (47%)	0 / 43 (0%)		5.9 (3.2 to 10)	
VEGFR-TKI > 18 mo	Nivo	0 / 30 (0%)	6 / 30 (20%)	15 / 30 (50%)	9 / 30 (30%)	0 / 30 (0%)		6.3 (4.6 to 8.6)	
**In 3rd line**	Cabo						0.46		0.4
VEGFR-TKI < 6 mo	Cabo	0 / 28 (0%)	11 / 28 (39%)	9 / 28 (32%)	4 / 28 (14%)	4 / 28 (14%)		13 (9.0 to 16)	
6 mo < VEGFR-TKI < 18 mo	Cabo	1 / 43 (2.3%)	20 / 43 (47%)	16 / 43 (37%)	4 / 43 (9.3%)	2 / 43 (4.7%)		11 (9.1 to 20)	
VEGFR-TKI > 18 mo	Cabo	0 / 30 (0%)	15 / 30 (50%)	13 / 30 (43%)	2 / 30 (6.7%)	0 / 30 (0%)		16 (14 to 30)	
**PFS $_{2\text{-}3}$**									0.2
VEGFR-TKI < 6 mo								18 (15 to 34)	
6 mo < VEGFR-TKI < 18 mo								24 (18 to 38)	
VEGFR-TKI > 18 mo								29 (23 to NE)	

[1]Fisher's exact test ; [2]Log-rank test

Cabo: cabozantinib; Nivo: nivolumab; VEGFR-TKI: vascular endothelial growth factor receptor tyrosine kinase inhibitor; CR: complete response; PR: partial response; SD: stable disease; PD: progressive disease; NE: not evaluable; mo: months; $PFS_{2\text{-}3}$: progression-free survival from 2nd line start to progression or death during 3rd line.

Caption: Best objective response and median progression-free survival (PFS) using RECIST 1.1 by investigator with nivolumab received in second line, cabozantinib received in third line, and nivolumab-cabozantinib sequence ($PFS_{2\text{-}3}$ only) according to duration of VEGFR-TKI treatment in first line.

Supplementary figures

Figure S1 – Propensity score distribution in overall population

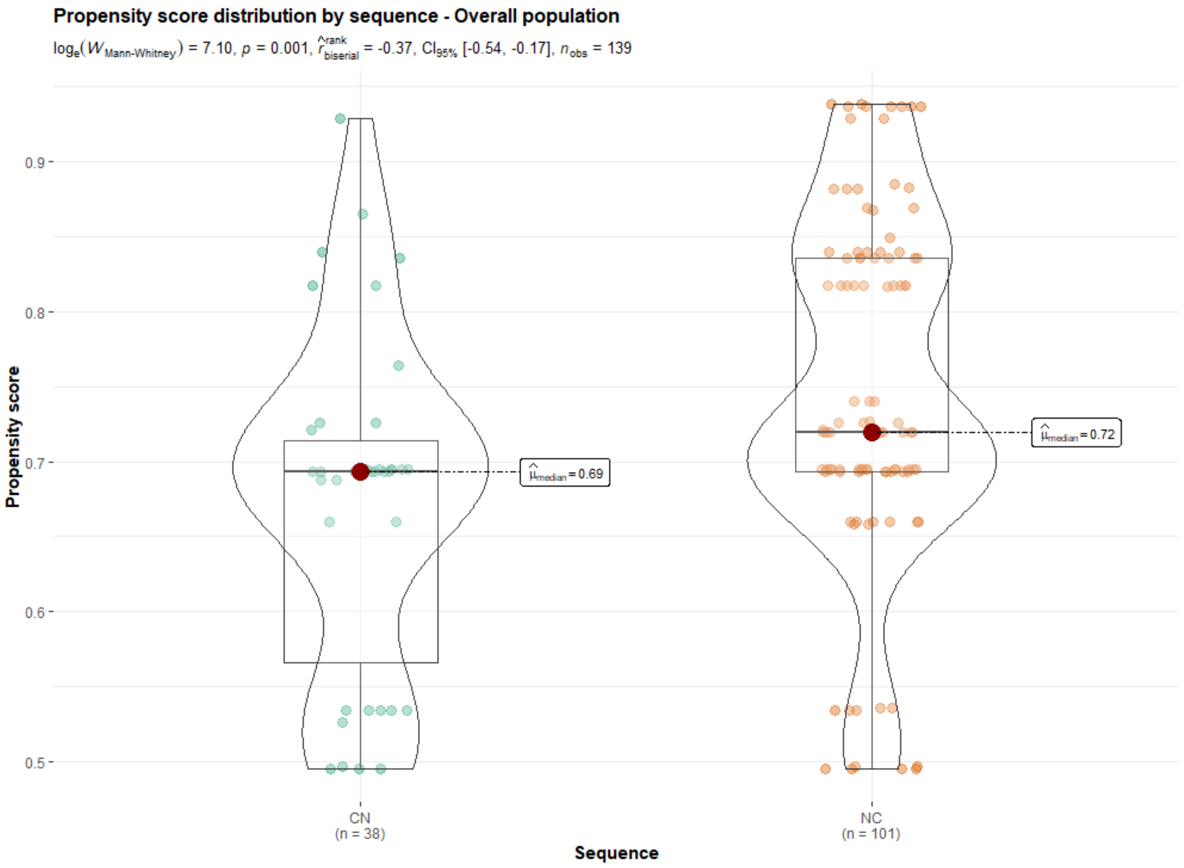

CN: cabozantinib-nivolumab sequence; NC: nivolumab-cabozantinib sequence ; PrS : propensity score.

Caption: Propensity score (PrS) is calculated from logistic regression. The distribution of PrS in each arm must have some overlapping values for the matching to be performed. Here, plots show that overlapping was sufficient to match each patient of the CN sequence with a patient of the NC sequence.

Figure S2 – Propensity score distribution in matched population

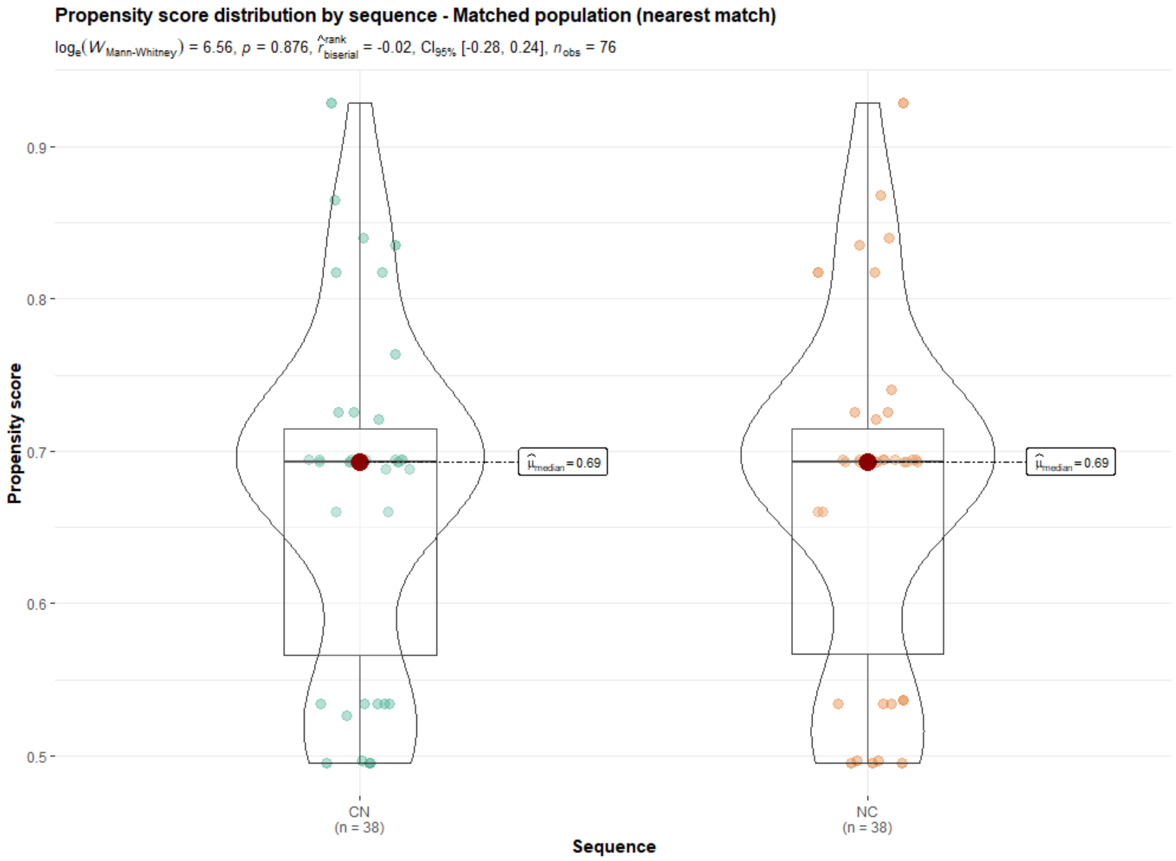

CN: cabozantinib-nivolumab sequence; NC: nivolumab-cabozantinib sequence; PrS : propensity score.

Caption: Plot showing distribution of propensity score (PrS) in each arm after matching. When matching is done on the PrS, baseline characteristics included in the PrS model should be balanced.

Figure S3 –Overall survival in 2nd line (OS_2) in matched 1:1 population

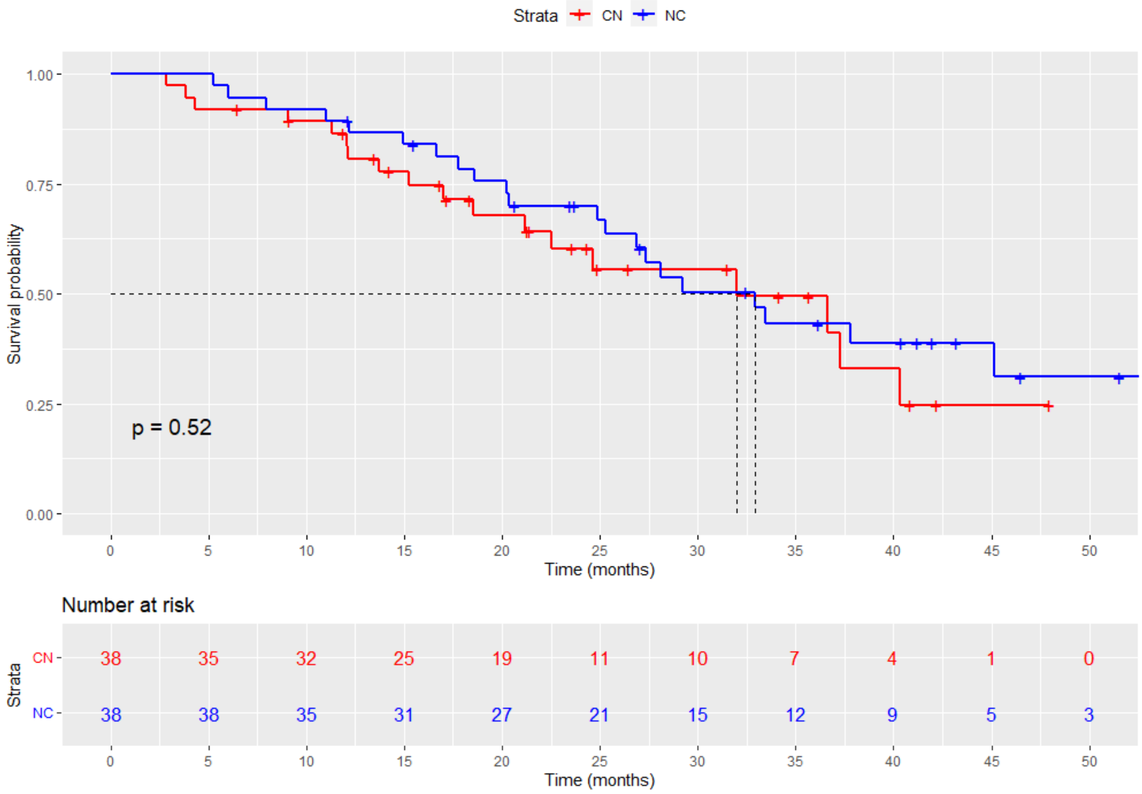

CN: cabozantinib-nivolumab sequence; NC: nivolumab-cabozantinib sequence.

<u>Caption:</u> Kaplan-Meier curve of overall survival from 2nd line in matched (1:1) population (n=38). The red curve represents the cabozantinib-nivolumab (CN) sequence and the blue curve represents the nivolumab-cabozantinib sequence.

Figure S4 –Overall survival in 2nd line (OS_2) by 1st line duration subgroups in matched 1:1 population

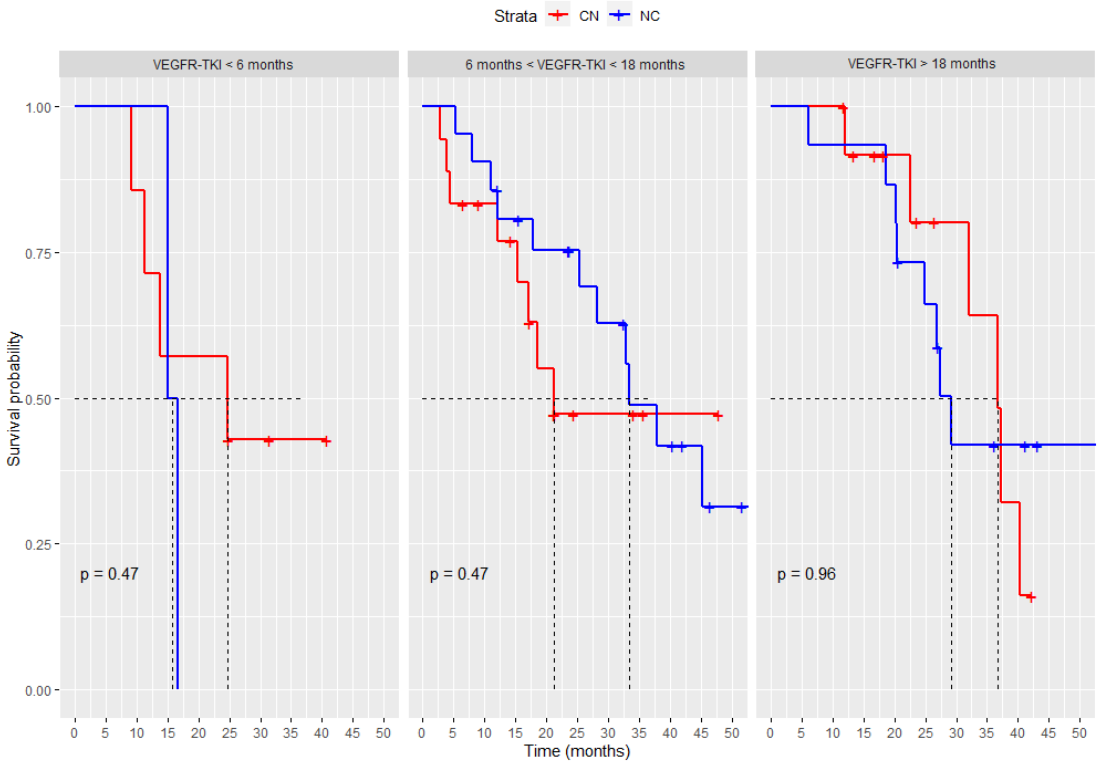

CN: cabozantinib-nivolumab sequence; NC: nivolumab-cabozantinib sequence.

<u>Caption</u>: Kaplan-Meier curve of overall survival from 2nd line in matched (1:1) population (n=38) according to duration of 1st line VEGFR-TKI treatment.

Left panel: 1st line duration < 6 months; middle panel: 1st line duration >6 months and <18 months; right panel: 1st line duration >18 months.

The red curve represents the cabozantinib-nivolumab (CN) sequence and the blue curve represents the nivolumab-cabozantinib sequence.
